# EXPLORING END-OF-LIFE DEMENTIA CARE IN RESIDENTIAL CARE SETTINGS: TOWARD A RELATIONAL MODEL

**DOI:** 10.1093/geroni/igae098.2387

**Published:** 2024-12-31

**Authors:** Pia Kontos, Sherry Dupuis, Romeo Colobong, Erin Delaney, Robin Gertin, Katia Engell, Erica MacTavish

**Affiliations:** KITE Research Institute, Toronto Rehabilitation Institute – University Health Network; Dalla Lana School of Public Health, University of Toronto, Toronto, Ontario, Canada; University of Waterloo, Waterloo, Ontario, Canada; KITE Research Institute, Toronto Rehabilitation Institute – University Health Network, Toronto, Ontario, Canada; University of Waterloo, Waterloo, Ontario, Canada; The Bitove Method, Toronto, Ontario, Canada; The Bitove Method, Toronto, Ontario, Canada; University of Waterloo, Waterloo, Ontario, Canada

## Abstract

With the biomedicalization of death in Canada, and the stigma associated with both dementia and death, people living with dementia (PLwD) in Canada suffer painful and undignified end-of-life (EOL) experiences. There is growing evidence that relational approaches, which value relationships as complex and dynamic connections including broader social, cultural, political, and environmental forces, can reduce stigma and improve quality of care. Yet, these approaches have not been explored or implemented in EOL care. Further, despite that EOL care is a priority in a number of national dementia strategies, EOL research in Canada remains limited and has largely neglected the inclusion of PLwD. To address these limitations, we conducted arts-based research conversations with PLwD in residential care settings in Ontario, Canada. These sessions were guided by Liberation Arts (i.e., using the arts to challenge oppressive attitudes, policies and practices), co-facilitated by community artists and researchers, and focused on what compassionate, relational EOL care looks like from the perspectives and experiences of PLwD. Transcripts of the audio-recorded conversations and the art created with PLwD were analyzed using the participatory Critical and Creative Hermeneutic Analysis Framework. Our analysis highlights EOL as relational (i.e., a communal rather than an individual process), as demonstrated by prioritizing relationships (e.g., human, pets, biographical objects, place/space), honoring PLwD (e.g., cultural/religious traditions, contributions to life), and supporting living life until the end. Our analysis contributes to the nascent EOL research that is inclusive of PLwD and will importantly inform a new model of EOL care grounded in relational caring.

